# Acute Calcific Retropharyngeal Tendinitis with Eggshell-like Calcification: Case Report and Literature Review on Time-course Changes in Imaging Findings

**DOI:** 10.7759/cureus.7611

**Published:** 2020-04-10

**Authors:** Norio Yamamoto, Takashi Watari, Keisuke Kawasaki, Yuzuru Matsui, Toshifumi Ozaki

**Affiliations:** 1 Department of Orthopedic Surgery, Unnan City Hospital, Unnan, JPN; 2 Department of Internal Medicine, Postgraduate Clinical Training Center, Shimane University Hospital, Izumo, JPN; 3 Department of Orthopedic Surgery, Kagawa Prefectural Central Hospital, Takamatsu, JPN; 4 Department of Orthopedic Surgery, Okayama University Graduate School of Medicine, Dentistry and Pharmaceutical Science, Okayama, JPN

**Keywords:** acute calcific retropharyngeal tendinitis, acute calcific tendonitis of the longus colli, eggshell calcification, time-course changes

## Abstract

Acute calcific retropharyngeal tendinitis is a rare disease, and few studies have reported the radiological findings of its time-course in detail. These radiological findings vary according to the calcific stage. We report a case of acute calcific retropharyngeal tendinitis with eggshell-like calcification detected on follow-up computed tomography (CT). We also review pertinent literature on calcific retropharyngeal tendinitis, with a focus on time-course changes in imaging findings.

A 54-year-old Japanese woman presented with acute severe neck pain. She also had a limited range of motion in the rotation of her neck and moderate pain and discomfort during swallowing. Plain radiographs of the cervical spine showed no apparent abnormality. CT revealed massive retropharyngeal calcification in front of the C1-C2 vertebrae. The patient was diagnosed with acute calcific retropharyngeal tendinitis and treated with a soft collar and non-steroidal anti-inflammatory drugs. Two weeks later, the neck pain and dysphagia improved. At the one-month follow-up, CT showed residual marginal calcification, which was diminishing in size, suggesting eggshell-like calcification.

We believe that although the eggshell calcification appearance is extremely rare, it is important to note this atypical presentation of acute calcific retropharyngeal tendinitis.

## Introduction

Acute calcific retropharyngeal tendinitis is a reactive, self-limiting inflammatory response to the acute deposition of calcium hydroxyapatite crystals in the tendons of the longus colli muscle at the anterior C1-C2 [1]. It is a rare disease, which is probably underdiagnosed because physicians are often unfamiliar with its presentation; further, it has a self-limiting pathology [2]. Although previous studies have described the radiological presentation on diagnosis, only a few reports have provided the radiological findings of its time-course in detail [3]. This disease is thought to be a self-limiting condition that resolves spontaneously after one to two weeks. Although symptoms are not always synchronous with their radiological findings, one to two months may be needed for complete healing on the basis of the literature review [3].

Herein, we report a case of acute calcific retropharyngeal tendinitis with eggshell calcification, which was detected on follow-up computed tomography (CT). We also review the pertinent literature on calcific retropharyngeal tendinitis, with a focus on time-course changes in imaging findings.

## Case presentation

Table 1 shows our review of the literature.

**Table 1:** Review of the literature on acute calcific retropharyngeal tendinitis with a focus on the time-course changes in the imaging finding F, female; M, male; F/U, follow-up; CT, computed tomography; MRI, magnetic resonance imaging

Authors	Age (years) / Sex	F/U image and period	F/U image findings	Symptomatic resolution
Tezuka et al. [3]	59 / F	CT, 3 weeks; MRI, 6 months	CT: almost complete disappearance of the amorphous calcification. MRI: no fluid in the retropharyngeal space.	Two weeks
Tamm et al. [4]	41 / F	CT, 2 months	Resolution of the effusion and near-complete resolution of the calcification.	Three months
Tagashira et al. [5]	40 / M	CT, 3 months	The calcification had disappeared.	Three days
Estimable et al. [6]	45/ M	MRI, 7 weeks	Improvement in the prevertebral non-enhancing walled fluid collection.	One week
Oh et al. [7]	25 / M	Radiograph, 2 months	The prevertebral swelling markedly improved.	Two months
Kim et al. [8]	41 / F	CT, 1 week	Decrease in the calcific deposit and retropharyngeal soft tissue swelling.	Ten days
Narváez et al. [9]	47 / F	CT, 5 months	Calcification and soft tissue swelling had disappeared.	Three months
Matsuura et al. [10]	44 / M	CT, 3 months	Calcification and soft tissue swelling had disappeared.	Four days
Present case	54 / F	CT, one month	Residual marginal calcification, decreasing in size.	Two weeks

A 54-year-old Japanese woman presented with acute severe neck pain. The pain was of acute onset and had started 12 hours prior to her hospital visit. She had no significant medical history. Her vital signs were normal, with the exception of mild fever (37.5°C). She also had a limited range of motion during rotation of her neck and moderate pain and discomfort during swallowing. She had no oropharyngeal abnormalities, meningeal signs, and neurological abnormalities.

**Figure 1:**
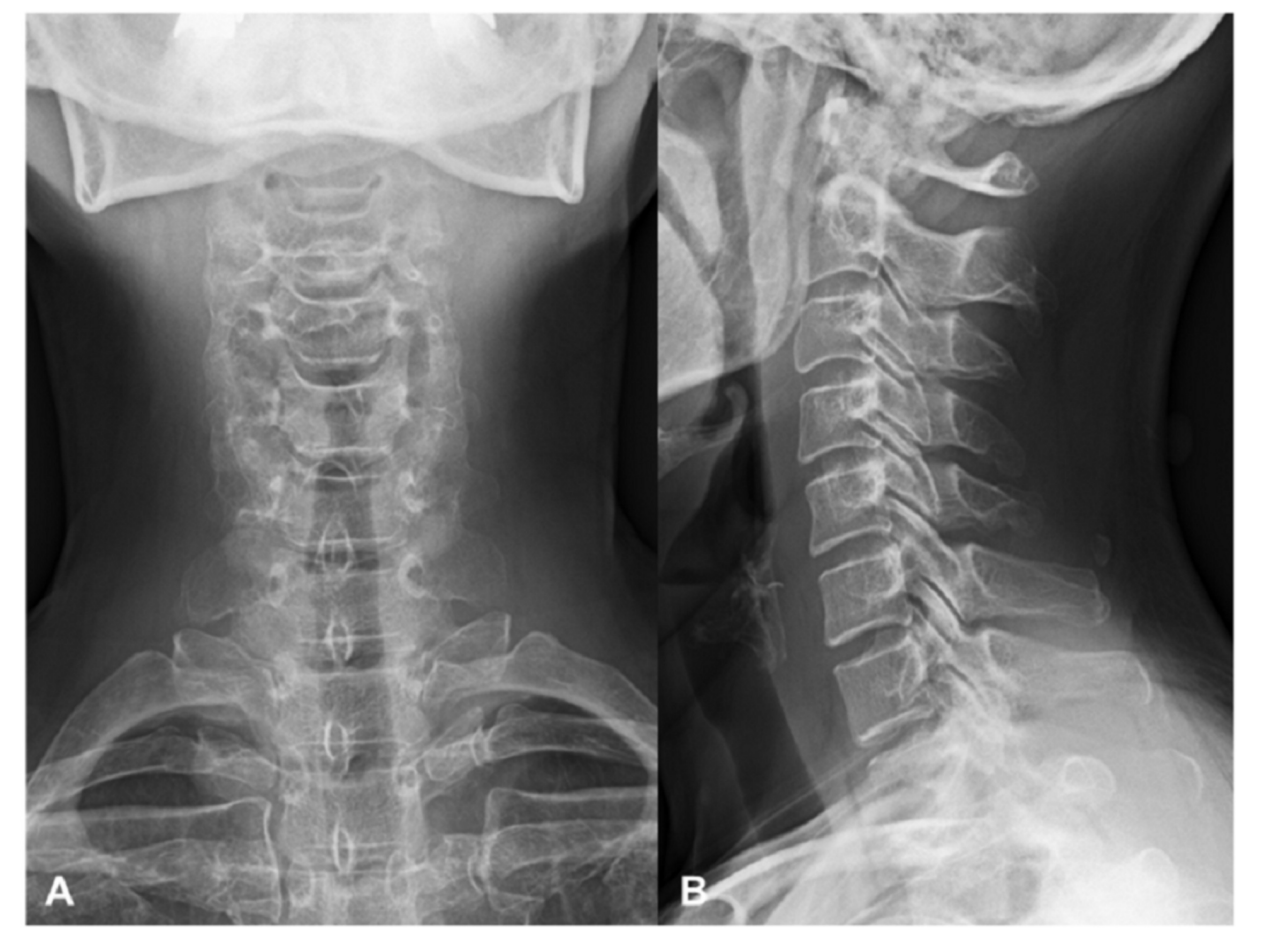
Plain radiographs of the cervical spine on initial presentation Anteroposterior (A) and lateral (B) radiographs showing no apparent abnormality

**Figure 2:**
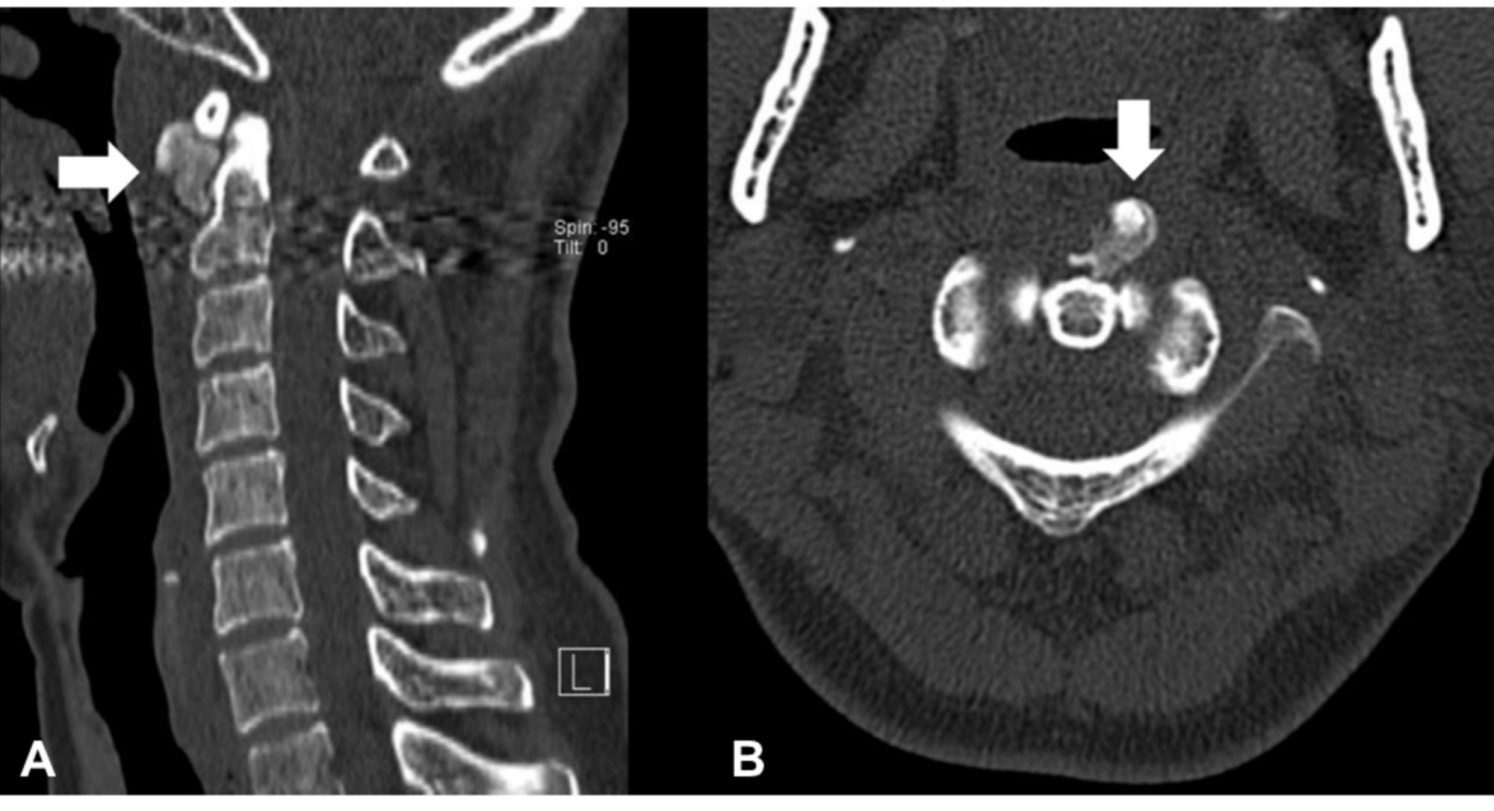
Non-contrast computed tomography (CT) scans of the cervical spine on initial presentation (A) Sagittal and (B) Axial CT scan (bone window) demonstrating amorphous calcification anterior to the C1 and C2 levels with the associated soft tissue swelling (arrows)

**Figure 3:**
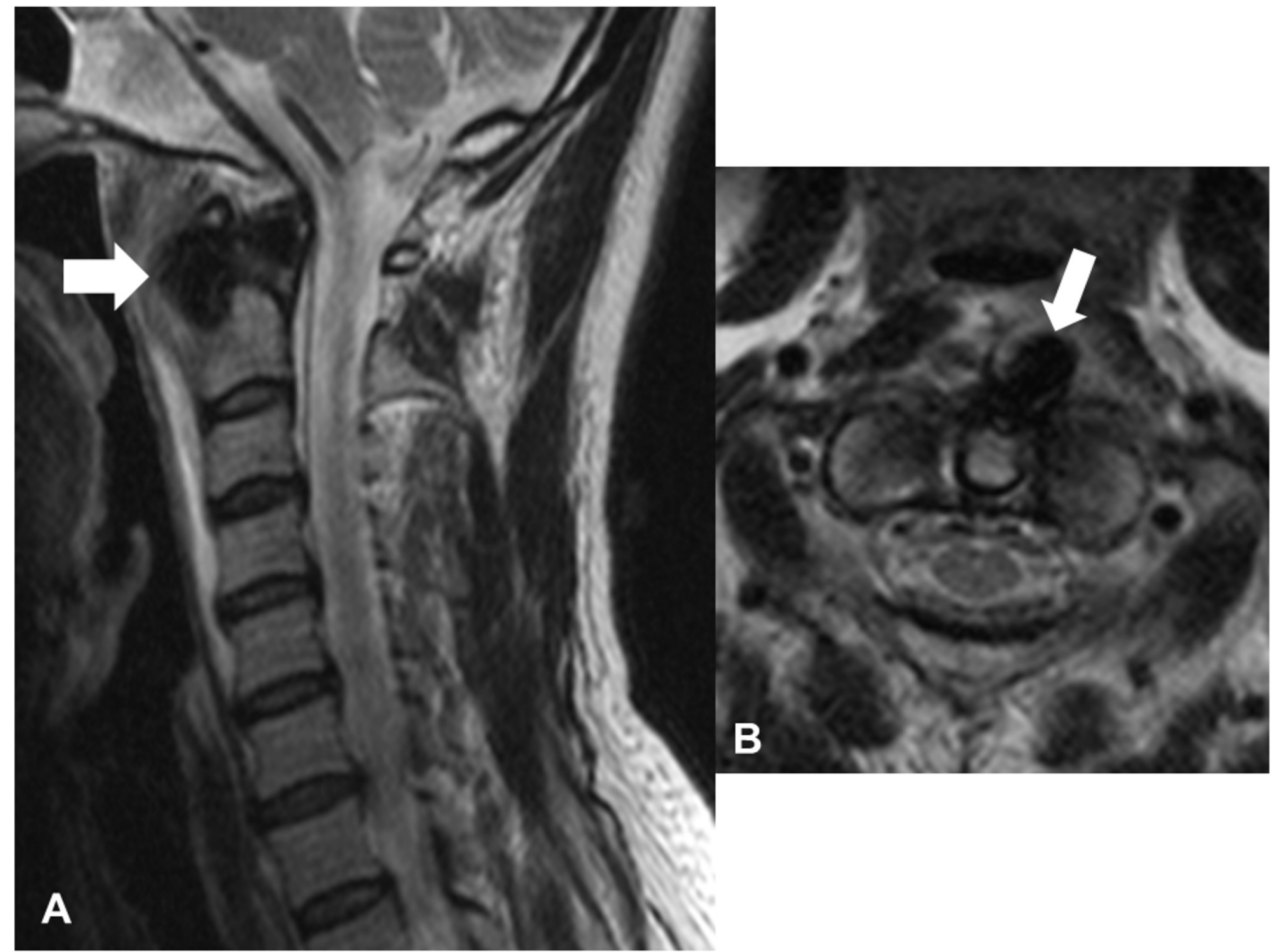
Magnetic resonance imaging of the cervical spine on initial presentation (A) Sagittal and (B) Axial T2-weighted images showing increased signal intensity in the retropharyngeal space between C1 and C5 (arrows)

Laboratory data showed a slight elevation of C-reactive protein (0.29), an erythrocyte sedimentation rate of 16 mm/h, and a normal white blood cell count (4100/ul). Other laboratory findings were within normal limits. Plain radiographs of the cervical spine showed no apparent abnormality (Figure 1). Owing to suspicion of emergent diseases, such as a retropharyngeal abscess, CT of the neck was performed. It revealed massive retropharyngeal calcification with its longest diameter (18 mm) in front of the C1-C2 vertebrae (Figure 2). It was inhomogeneous and irregularly shaped with associated soft tissue swelling. Magnetic resonance imaging (MRI) showed markedly increased signal intensity on T2-weighted images and intermediate signal intensity on T1-weighted images at the anterior C1 to C5 level (Figure 3). There were no findings of spondylitis, epidural abscess, or tumor.

**Figure 4:**
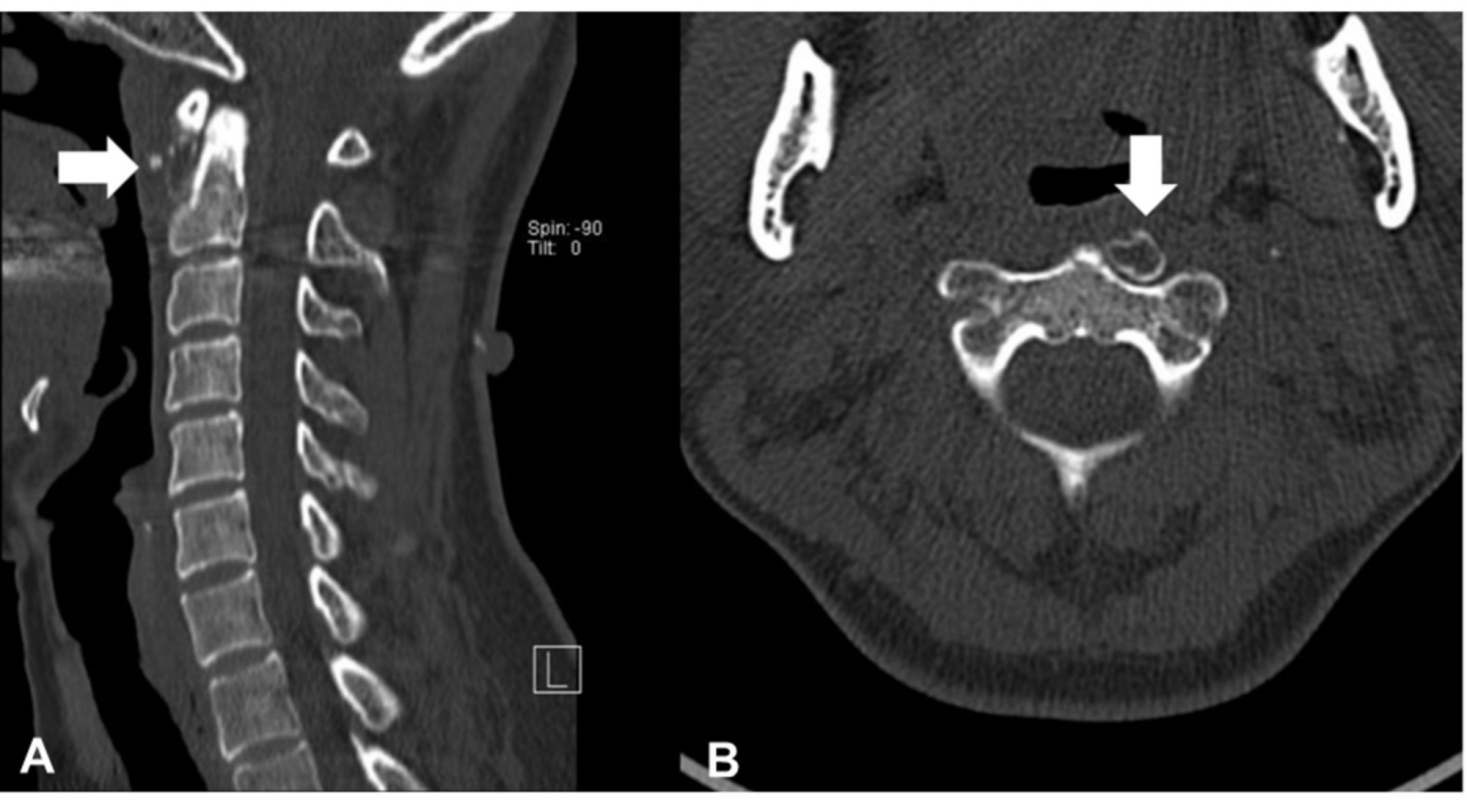
Computed tomography (CT) scans of the cervical spine at the one-month follow-up (A) Sagittal and (B) Axial CT showing marginal calcification, suggesting an eggshell-like calcification with reduced soft tissue swelling (arrows)

Thus, the patient was diagnosed with acute calcific retropharyngeal tendinitis, and the condition was treated with a soft collar and non-steroidal anti-inflammatory drugs (NSAIDs). Two weeks later, the neck pain and dysphagia improved. At the one-month follow-up, she had a complete resolution of the symptoms. A follow-up CT showed residual marginal calcification, which was diminishing in size, suggesting an eggshell-like calcification (Figure 4).

## Discussion

To our knowledge, this is the first report of acute calcific retropharyngeal tendinitis with eggshell calcification detected on follow-up CT. A few cases on acute calcific retropharyngeal tendinitis, including the time-course changes in the imaging findings, have been reported (Table 1) [3-10]. All cases in the literature review showed symptomatic resolution and improvement of the acute calcific retropharyngeal inflammation on consecutive images, although the previous studies reported that it was a self-limiting disease without follow-up imaging findings. Residual marginal calcification was only reported in our case. We speculate that the time-course differences in imaging findings may be attributed to the degree of inflammation and the intervals at which consecutive images were obtained.

The pathogenesis of the calcium deposition was suspected to be related to trauma, repetitive ischemia, and degeneration. The natural history of acute calcific retropharyngeal tendinitis according to image findings remains unclear. Calcific tendinitis has the following three time-course stages: pre-calcification, calcific, and post-calcific [11]. During the resorptive phase of the calcific stage, calcific deposits are invaded by macrophages, polymorphonuclear cells, and fibroblasts. This is the most painful phase of calcific tendonitis, and the calcium deposits have a toothpaste-like appearance. Subsequently, the post-calcific stage is characterized by granulation tissue formation, which replaces the space that was previously occupied by the calcium deposits [11]. A previous report on the radiologic course of acute symptomatic calcific tendinopathy of the shoulder indicated that 46% of the calcific deposits tended to become cloudier and more inhomogeneous than the initial findings, and 62% of cases presented with complete resolution or decrease in size [12]. In the present case, we speculated that the eggshell calcification phenomenon might be a marginal deposit at the post-calcific stage, although symptoms are not synchronous with the radiological findings.

The time required for complete absorption of the calcium deposits was about one month [3]. In the clinical setting, when the CT finding of a patient with neck pain shows eggshell calcification, clinicians should suspect calcific retropharyngeal tendinitis at the post-calcific stage and should avoid unnecessary interventions. The differential diagnosis of eggshell calcification includes esophageal schwannoma, thyroid tumor, retropharyngeal abscess, lymph node calcification, heterotopic ossification, and ossifying myositis [13-14].

## Conclusions

The radiological findings of the time-course of acute calcific retropharyngeal tendinitis show different findings according to the calcific stage. Although the eggshell-like calcification appearance is extremely rare, it is important to note this atypical presentation of acute calcific retropharyngeal tendinitis.
